# Mastoiditis With Pneumocephalus on CT: An Uncommon Clue to Underlying Bacterial Meningitis

**DOI:** 10.7759/cureus.103191

**Published:** 2026-02-08

**Authors:** Meera Alshehhi, Hafidh Alrajaby, Lamiz Tannouri

**Affiliations:** 1 Emergency Department, Rashid Hospital, Dubai Health, Dubai, ARE; 2 Emergency Medicine Department, Rashid Hospital, Dubai Health, Dubai, ARE

**Keywords:** bacterial meningitis, mastoiditis, non-traumatic pneumocephalus, pneumocephalus, streptococcus pneumoniae meningitis

## Abstract

The combination of pneumocephalus and meningitis as a complication of mastoiditis is rare, with very few cases published in the literature. We report the case of a 33-year-old previously healthy male who presented with acute confusion, fever, agitation, and neck stiffness following a syncopal episode at work. CT imaging revealed opacification of the left mastoid air cells, suggestive of mastoiditis and pneumocephalus in the left frontoparietal region. Cerebrospinal fluid (CSF) analysis confirmed the diagnosis of pneumococcal meningitis. Empirical antibiotic treatment and steroids were initiated, and the patient survived without any neurological complications or deficits.

## Introduction

Bacterial meningitis remains a life-threatening condition that requires immediate recognition and treatment. *Streptococcus pneumoniae* is the most common etiologic agent in adults, and although hematogenous dissemination is the typical route, local extension from infections such as otitis media or mastoiditis can also lead to central nervous system (CNS) involvement. Patients usually present with the classic triad of symptoms for meningitis: fever, neck stiffness, and altered mental status. However, all three are only present in 41% of cases of bacterial meningitis, and they are mostly seen in the elderly [1-3].

Pneumocephalus, defined as the presence of air within the cranial cavity, is an uncommon radiological finding in non-traumatic settings. Its presence in the absence of prior surgery or trauma should prompt evaluation for infectious etiologies, including meningitis with dural involvement [1]. This report describes an unusual presentation of pneumococcal meningitis with imaging evidence of spontaneous pneumocephalus and mastoiditis in a young adult.

## Case presentation

**Figure 1:**
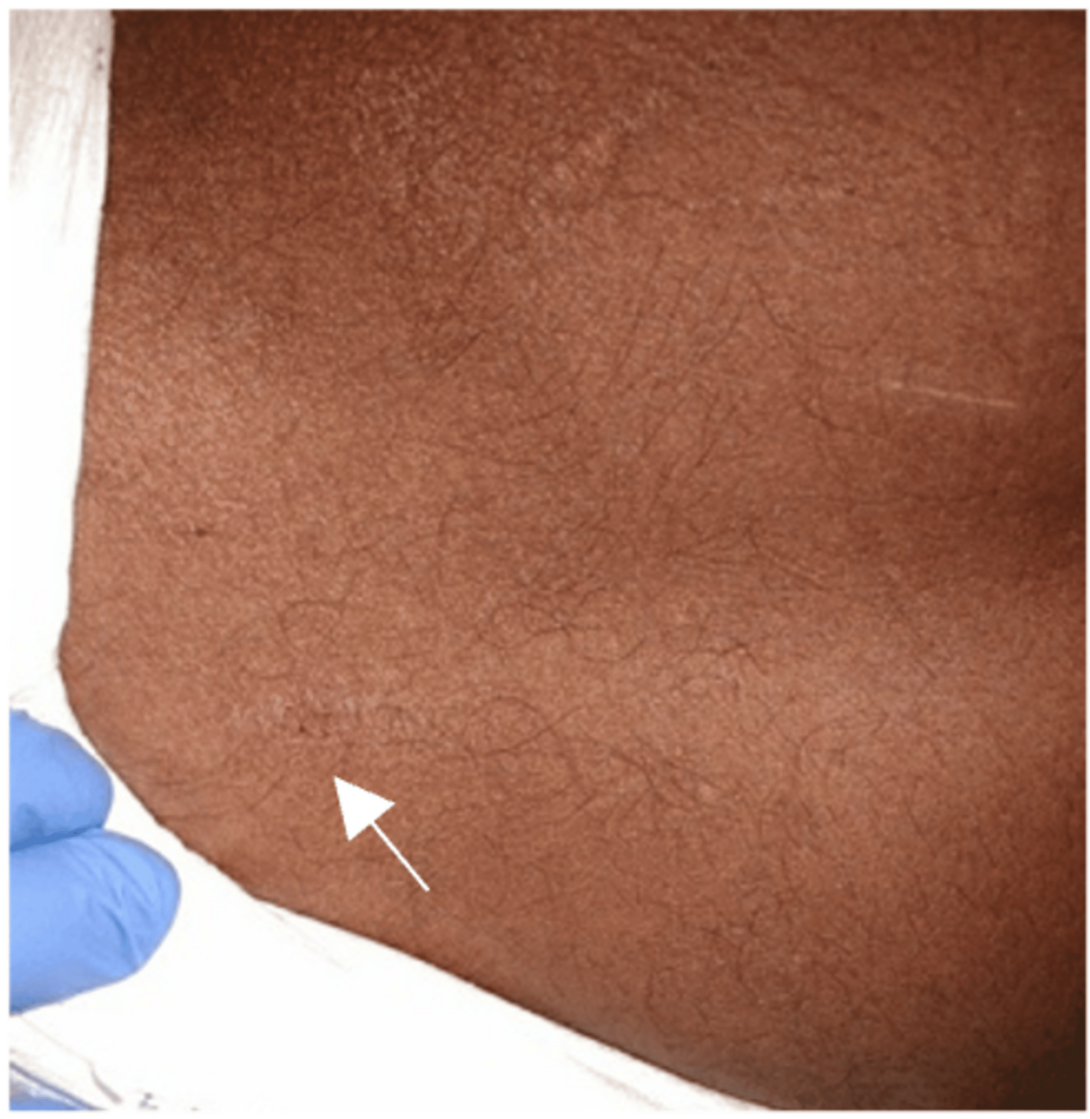
Abrasion noted over the mid lower back

**Figure 2:**
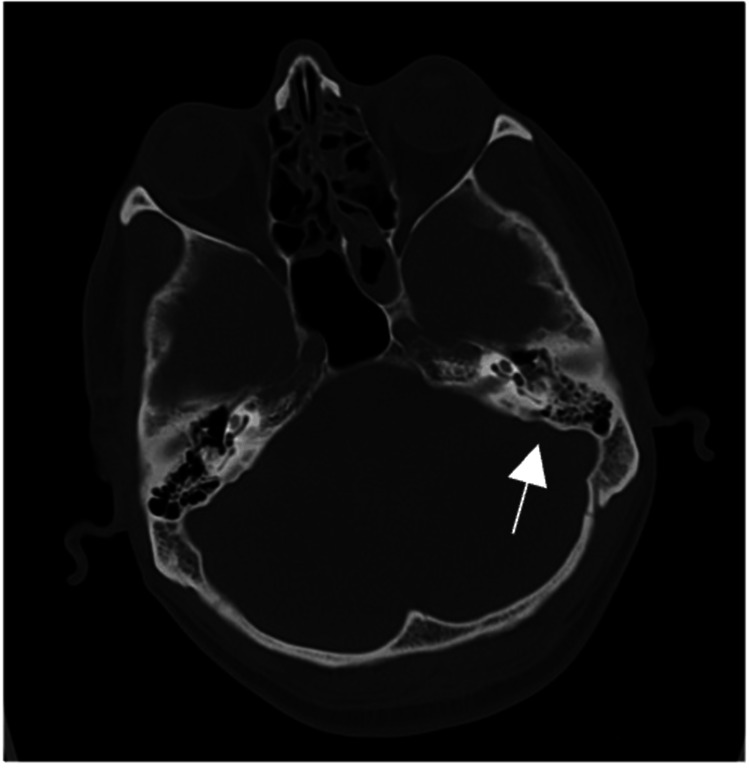
Axial view of brain CT showing opacification left mastoid air cells, possibly related to old mastoiditis or trauma

**Figure 3:**
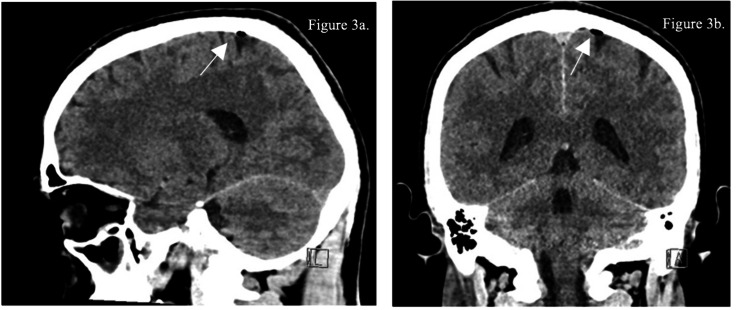
(a) Sagittal view of brain CT and (b) coronal view of brain CT showing air vacuole in the left frontoparietal region

A 33-year-old previously healthy male presented to the Emergency Department (ED) with a one-day history of confusion, poor verbal responses, and abnormal behavior, preceded by a two-day history of fever, cough, poor appetite, and one episode of sudden dizziness with loss of consciousness at work. After he regained consciousness, he appeared agitated and unaware of his surroundings. Upon arrival at the ED, the patient was febrile (38.2°C), tachycardic, tachypneic, and agitated. He appeared ill and disoriented, with a Glasgow Coma Scale (GCS) score of 13/15 (eye opening: 4, verbal response: 4, motor response: 5) and pupils of 3 mm reactive and equal bilaterally, and neck stiffness was noted on examination. When examining the skin, the patient had an abrasion over the mid lower back (Figure 1). The patient's relative denied any procedures that were done in the previous facilities, including a lumbar puncture (LP). When checking our integrated system with other facilities that show some reports of medication, labs, and procedures that might have been done, there was no mention of any procedures done. There was no evidence of purpura or any other skin lesions, while the rest of the systemic examination was unremarkable. Initial investigations revealed elevated inflammatory markers (Table 1), and a computed tomography (CT) of the brain scan showed an air vacuole in the left frontoparietal region (Figure 2) and opacification of the left mastoid air cells, possibly related to old mastoiditis or trauma. No definite fracture lines or traumatic brain injury were seen (Figure 3). 

**Table 1:** Results of inflammatory markers over the period of hospital stay

Hospital Day	Day 1	Day 2	Day 4	Day 8	Day 14	Reference Range
WBC Count	24.2	25.8	10.7	9.5	12.5	10 units, 3/uL
C-reactive Protein	117.2 mg/L	451.6 mg/L	86.2 mg/L	6.7 mg/L	2.1 mg/L	<5.0 mg/L
Procalcitonin	6.08 ng/mL	6.14 ng/mL	1.09 ng/mL	0.14 ng/mL	0.06 ng/mL	<0.05 ng/mL

Four attempts of LP in the ED resulted in dry taps. The patient was started on ceftriaxone, dexamethasone, and intravenous (IV) fluids, and airborne and droplet precautions were initiated. He was admitted under the infectious disease unit with a working diagnosis of suspected bacterial meningitis. While awaiting the results of a full septic workup, he was continued on vancomycin, ceftriaxone, and dexamethasone. A fluoroscopically guided LP was later attempted by the interventional radiology team. During his hospital stay, the patient showed significant improvement with resolution of agitation and improved orientation, although he continued to report mild headache and persistent neck stiffness. Cerebrospinal fluid (CSF) analysis (Table 2) revealed a high WBC count (900 cells/μL, 90% polymorphs), elevated protein, normal glucose, and Gram-positive cocci on Gram stain. CSF culture and blood cultures showed no growth after five days. *S. pneumoniae* was detected by polymerase chain reaction (PCR) testing of the CSF as the causative organism, and an MRI of the brain done on day three of admission showed left mastoiditis without signs of meningoencephalitis. The patient was diagnosed with acute bacterial meningitis secondary to *S. pneumoniae *and completed a 14-day course of high-dose ceftriaxone, vancomycin, dexamethasone, and supportive care. He was vaccinated with the pneumococcal conjugate vaccine (Prevenar 13) before discharge and advised to receive the pneumococcal polysaccharide vaccine (Pneumovax 23) after eight weeks. 

**Table 2:** Cerebrospinal fluid analysis results

Results		Reference Range
WBC count	900 cells/ μL, 90% polymorphs.	0-5 cells/ μL
Protein	92 mg/dL	15-45 mg/dL
Glucose	70 mg/dL	40-76 mg/ dL
Lactic acid	5.06 mmol/L	1.1-2.4 mmol/L
Gram stain	Gram-positive cocci in pairs, most probably pneumococci	No organism seen

## Discussion

Pneumocephalus is defined as the presence of air within the cranial cavity, most commonly encountered in the setting of head trauma, neurosurgical interventions, tumours, radiation necrosis, and meningitis caused by gas-forming organisms [1,2,4]. Usually, pneumocephalus is asymptomatic, but signs and symptoms are variable, ranging from confusion and altered mental status to headache, vomiting, and seizures [4,5].

It is a rare complication of pneumococcal meningitis, and in these cases, the occurrence of pneumocephalus has been reported in patients with otitis media, sinusitis, and mastoiditis [4,5]. Its occurrence in a previously healthy adult without recent trauma or surgery is exceedingly rare and should raise concern for underlying infection, particularly with possible intracranial extension.

When it comes to the diagnosis of pneumocephalus the use of a computed tomography (CT) scan is preferred over the use of magnetic resonance imaging (MRI), as it can detect even 0.55 ml of intracranial air, whereas the presence of air in MRI may be perceived wrongly for flow voids or blood products [4,6], In our case, a CT scan revealed both pneumocephalus and opacification of the left mastoid air cells, suggesting a potential otogenic source. This raised suspicion of direct intracranial spread of infection from chronic or subclinical mastoiditis. Though uncommon, several case reports have documented bone erosion in mastoiditis, facilitating communication between the middle ear or mastoid and the intracranial space. This can lead to dural defects and subsequent CNS infection [4]. Taveira et al. reported a similar presentation of meningitis-associated pneumocephalus in a 44-year-old male, where his initial CT showed pneumocephalus that resolved after 48 hours of initiation of antimicrobial therapy, similar to our patient [1].

Timely empirical therapy is critical in the management of bacterial meningitis. Our patient was started on ceftriaxone, vancomycin, and dexamethasone at presentation, in line with established guidelines [4,7]. Early administration of dexamethasone, particularly in suspected or confirmed pneumococcal meningitis, has demonstrated benefit in reducing neurological complications such as hearing loss and long-term cognitive impairment. The delay in establishing a diagnosis and initiation of broad-spectrum antibiotics has been linked to increased mortality [6,7].

Treatment of pneumocephalus depends on the clinical condition of the patient and the degree and progression of air collection, in addition to addressing the etiology. Pneumocephalus itself is usually benign, and intracranial air is absorbed in 85% of patients during the first week [8].

Small pneumocephalus, secondary to meningitis or trauma, can be managed conservatively by treating the meningitis, monitoring the patient's neurological status closely, and repeat imaging to check for resolution. Large pneumocephalus with a midline shift requires immediate surgery in order to prevent herniation of the brain [9].

In suspected acute meningitis cases, CSF analysis is crucial, assessing white blood cell count, protein, glucose concentrations, and the CSF-to-blood glucose ratio. An LP should ideally occur before antibiotic treatment unless contraindicated by conditions such as bleeding risks, raised intracranial pressure, skin or soft tissue infection, or a suspected spinal epidural abscess overlying the LP site and hemodynamic or respiratory compromise.

Abnormal CSF findings, such as increased WBC concentration or protein levels above 45 mg/dL, indicate inflammation, while a CSF-to-blood or serum glucose ratio below 0.4 is considered low and indicates bacterial, fungal, or tuberculous meningitis. Lactate levels in CSF can help differentiate bacterial from viral meningitis [10].

Inflammatory markers, such as CRP and procalcitonin, were used to monitor disease activity. Elevated procalcitonin has been shown to reliably differentiate bacterial from viral CNS infections and serves as a valuable marker for tracking treatment response [10,11]. In our patient, these markers correlated well with clinical improvement, as shown in Table 1.

Blood cultures should be obtained early, preferably before starting antibiotics, as the diagnostic yield of blood culture may be substantially reduced when blood culture is obtained after the initiation of antibiotic therapy, especially in pneumococcal meningitis [10].

In suspected cases of acute meningitis, routine cranial imaging such as CT of the brain is not recommended. However, if there are signs of cerebral space-occupying lesions with midline shift, a Glasgow Coma Score (GCS) of less than 10, focal neurological signs, cranial nerve deficits, papilledema, new-onset seizures in adults, or a severe immunocompromised state, CT brain imaging should be done prior to LP [11].

Finally, in accordance with public health recommendations for patients recovering from invasive pneumococcal disease, the patient received the 13-valent pneumococcal conjugate vaccine (PCV13) prior to discharge, with plans for administration of the 23-valent pneumococcal polysaccharide vaccine (PPSV23) after eight weeks. This dual vaccination strategy is essential for long-term protection against pneumococcal disease as per the Center of Disease Control (CDC) guidelines. Adults aged ≥65 years who have not previously received pneumococcal vaccine or whose previous vaccination history is unknown should receive a dose of PCV13 first, followed by a dose of PPSV23. The dose of PPSV23 should be given 6-12 months after a dose of PCV13 [12].

## Conclusions

This case underscores the need for high clinical suspicion and early empiric therapy in patients presenting with signs of meningitis, especially when imaging reveals unusual findings such as pneumocephalus. Spontaneous pneumocephalus should prompt investigation for infectious sources, particularly mastoiditis, even in the absence of otologic symptoms. Timely antibiotic administration, advanced imaging, and interventional diagnostics can significantly improve patient outcomes. Pneumococcal vaccination remains a key preventive measure following recovery from invasive disease.
